# An Integrated Transcriptomic and Meta-Analysis of Hepatoma Cells Reveals Factors That Influence Susceptibility to HCV Infection

**DOI:** 10.1371/journal.pone.0025584

**Published:** 2011-10-25

**Authors:** Jamie I. MacPherson, Ben Sidders, Stefan Wieland, Jin Zhong, Paul Targett-Adams, Volker Lohmann, Perdita Backes, Oona Delpuech-Adams, Francis Chisari, Marilyn Lewis, Tanya Parkinson, David L. Robertson

**Affiliations:** 1 Computational and Evolutionary Biology, Faculty of Life Sciences, University of Manchester, Manchester, United Kingdom; 2 Pfizer Global Research and Development, Sandwich, United Kingdom; 3 The Scripps Research Institute, La Jolla, California, United States ofAmerica; 4 Department of Infectious Diseases, Molecular Virology, University of Heidelberg, Heidelberg, Germany; Tulane University, United States of America

## Abstract

Hepatitis C virus (HCV) is a global problem. To better understand HCV infection researchers employ in vitro HCV cell-culture (HCVcc) systems that use Huh-7 derived hepatoma cells that are particularly permissive to HCV infection. A variety of hyper-permissive cells have been subcloned for this purpose. In addition, subclones of Huh-7 which have evolved resistance to HCV are available. However, the mechanisms of susceptibility or resistance to infection among these cells have not been fully determined. In order to elucidate mechanisms by which hepatoma cells are susceptible or resistant to HCV infection we performed genome-wide expression analyses of six Huh-7 derived cell cultures that have different levels of permissiveness to infection. A great number of genes, representing a wide spectrum of functions are differentially expressed between cells. To focus our investigation, we identify host proteins from HCV replicase complexes, perform gene expression analysis of three HCV infected cells and conduct a detailed analysis of differentially expressed host factors by integrating a variety of data sources. Our results demonstrate that changes relating to susceptibility to HCV infection in hepatoma cells are linked to the innate immune response, secreted signal peptides and host factors that have a role in virus entry and replication. This work identifies both known and novel host factors that may influence HCV infection. Our findings build upon current knowledge of the complex interplay between HCV and the host cell, which could aid development of new antiviral strategies.

## Introduction

Hepatitis C virus (HCV) is prevalent in approximately 3% of the human population, though some countries, e.g., Eygpt, have a much greater prevalence [1]. The acute phase of infection is often asymptomatic whereas chronic infection is a major cause of liver cirrhosis, hepatocellular carcinoma and liver transplantation. Unfortunately there is no HCV vaccine. A high level of virion production, combined with the error-prone HCV RNA polymerase, causes frequent mutation of the viral genome resulting in production of immune escape mutants. Treatment of chronic HCV infection is currently based on interferon-α that evokes a general antiviral response and ribavirin, a nucleoside analogue. In combination, these antiviral agents do not reliably eradicate HCV in infected patients [2] and, in addition, treatment is often interrupted due to the side effects that these drugs cause [3]. Therefore, development of improved anti-HCV drugs would be of great benefit.

Drugs that bind specific host proteins essential to the virus life cycle pose an attractive approach in viral disease therapy, as these targets have less potential for mutation and associated emergence of resistance than viral protein targets. Thus, anti-HCV drugs that bind specific host proteins are currently in development. For example alisporivir, a Cyclophilin A inhibitor, has recently entered phase II trials [4]. Cyclophilin A is essential for efficient HCV replication, probably due to direct physical interaction with NS5A and mediation of the viral polymerase [5], . Also, inhibitors to microRNA mir-122, a molecule that regulates production of infectious virus particles, are also being investigated [7], [8]. By developing a greater understanding of the complex interplay between HCV and host cells, novel drug targets might be identified.

Significant advances in *in vitro* model systems to study HCV-host interactions have been made in the recent past [9]. Model systems greatly accelerated HCV research and led to production of a variety of genome-scale data sets including a host-virus interaction network [10], infection-induced changes in gene expression [11], [12] and host factors required for viral replication [5]. In addition to large data sets, numerous small-scale studies that use *in vitro* model systems have captured important details of key viral processes, such as virus cell entry [13]. However, we are still some considerable way from fully understanding the HCV life cycle and the role for each implicated host factor.

The initial breakthrough in HCV model systems allowed study of genomic viral RNA replication *in vitro* using replicons and permissive Huh-7 hepatoma cell lines [14], . More recent HCV cell-culture (HCVcc) systems have permitted study of the entire virus life cycle and rapid cell-to-cell transmission, using a specific combination of the JFH-1 HCV strain and a particularly permissive hepatoma cell line (Huh-7.5.1) [9], [16]. The Huh-7.5.1 cells have a deactivating mutation in retinoic acid-inducible gene I (RIG-I), a protein that would normally bind to HCV RNA and initiate an interferon based antiviral response in the cell [17], [18]. Also, further subcloning of Huh-7.5.1 has led to the production of a more permissive cell (designated Huh-7.5.1c2 [19]), though the underlying mechanism of increased permissiveness in this subclone is not understood.

In addition to HCV susceptible cells, infection resistant Huh-7 derived cells have also been produced. One HCVcc study by Zhong *et al*. [20], a prerequisite to this study, detected coevolution of JFH-1 HCV virus and Huh-7 and Huh-7.5.1 derived host cells. In particular, evolution of an increasingly aggressive virus was associated with emergence of several resistant cells. Follow-up analysis revealed that reduced cell surface expression of the CD81 viral coreceptor [21], [22] was partly responsible for resistance in a subset of these cells and that additional defects must be present that perturb the viral life-cycle. Therefore, mechanisms of both HCV resistance and susceptibility for Huh-7 derived cells are yet to be determined. This knowledge will be valuable for understanding specific host-cell dependencies in the viral life cycle and developing novel antiviral strategies.

**Figure 1:**
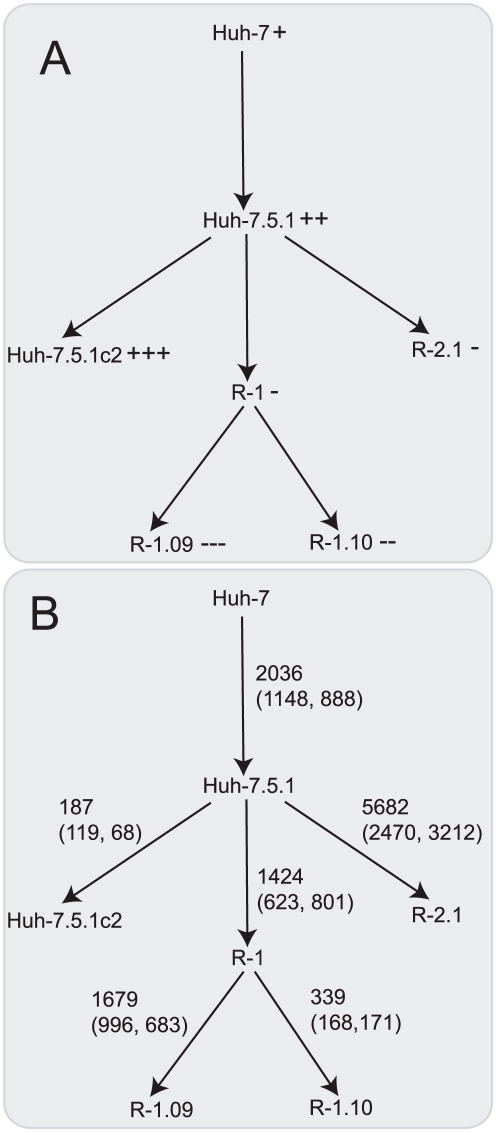
Tree of hepatoma cell cultures. Cell cultures are joined by arrows, going from the parent to the descendent, that indicate a subcloning event (or in the case of Huh-7 to Huh-7.5.1 a series of subcloning events). (A) The relative susceptibility of these cells to HCV infection where “+” represents susceptibility and “-” represents resistance and more symbols represent greater susceptibility or resistance. (B) Differential expressed genes between subclones. Differentially expressed genes were assigned to this tree either directly from expression comparison between cells or indirectly using a parsimony method. On each arrow, the first number indicates the total number of differentially expressed genes that have been attributed to the subcloning event. Below in brackets are the number of these genes that are (i) downregulated or (ii) upregulated following subclononing.

In order to elucidate mechanisms by which hepatoma cells are susceptible or resistant to HCV infection we performed genome wide expression analysis of six Huh-7 derived cell cultures that have different levels of permissiveness to infection (figure 1A). To focus our investigation, we identified host proteins from HCV replicase complexes that were present in small vesicles located in the membranous web – a specific membrane alteration that is the site of HCV replication [23] – and also performed gene expression analysis of three permissive HCV infected cells. We found that a great number of genes, representing a wide spectrum of functions, including factors known to be involved in viral entry were differentially expressed between cells with different permissiveness to infection. Following this we conducted an in-depth analysis of differentially expressed host factors by integrating multiple data sources. Using this approach, we demonstrate that changes relating to susceptibility to HCV infection can be specifically linked to the innate immune response, secreted signal peptides, known host factors that influence virus entry and replication and putative, novel HCV infection-related host factors. In addition, our study also helps to characterise Huh-7 derived cells which may aid interpretation of results from subsequent studies that use HCVcc.

## Results and Discussion

### HCV infection causes significant changes to gene expression

**Figure 2:**
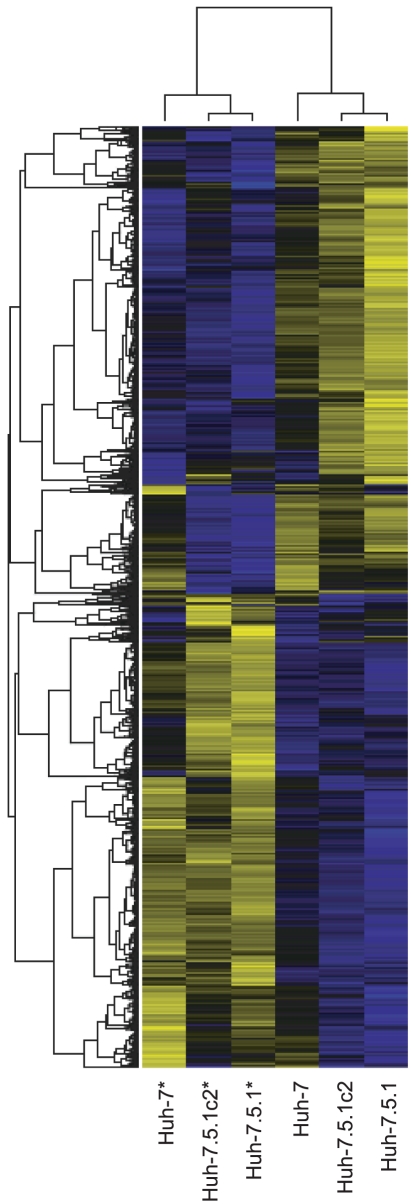
Hierarchical clustering plot displaying differentially expressed genes from infected and control cells. Genes are represented by horizontal bands and cells by columns. Infected cells are denoted with an asterisk (*). Bands are coloured blue if the gene is downregulated and yellow if it is upregulated compared with the mean expression level for that gene. Greater colour intensity signifies greater fold change. Infected cells cluster with one another and gene clustering shows a clear pattern that corresponds to HCV infection-induced regulation of gene expression.

**Figure 3:**
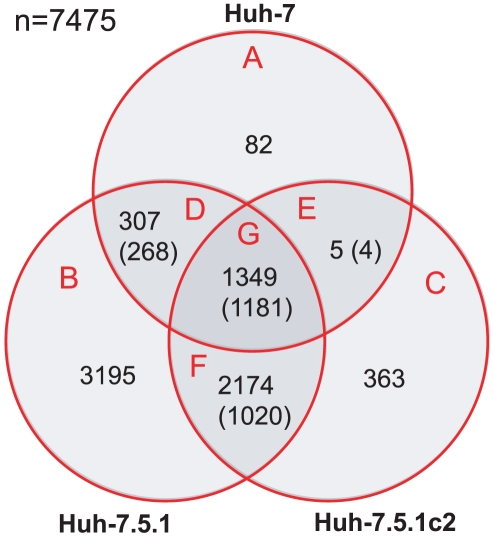
Venn diagram showing the overlap in genes differentially expressed due to HCV infection among susceptible cells. The absolute numbers of significantly differentially expressed genes are given for Huh-7, Huh-7.5.1 and Huh-7.5.1c2 cells. The numbers in brackets refer to those genes that share the same direction of regulation (up- or downregulated) following infection, across multiple comparisons.

Expression analysis of three hepatoma cell cultures – Huh-7, Huh-7.5.1 and Huh-7.5.1c2 – that are susceptible to infection was performed. By comparing infected and uninfected cells we identified genes that are differentially expressed (false discovery rate corrected p-value <0.01), in all cell lines: 1743 genes in Huh-7 (1525 upregulated, 218 downregulated); 7025 in Huh-7.5.1 (3503 upregulated, 3522 downregulated); and 3891 in Huh-7.5.1c2 (1485 upregulated, 2406 downregulated), (supplementary table S1). This represents 7475 distinct genes, 54% of all genes analysed. 3835 of these genes (51%) were differentially expressed in more than one cell and 1181 (16%) were differentially expressed with the same response to infection (up- or downregulation) in all three comparisons. A hierarchical clustering plot (figure 2) shows a clear pattern of gene expression that corresponds to infection by HCV. Intersections between these gene sets are shown in figure 3.

Previously, Woodhouse *et al.* [24] performed whole genome expression analysis of Huh-7.5 cells infected with JFH1 HCV, harvested at the peak of infection and identified 1351 differentially expressed genes. Though we identify more differentially expressed genes, our results overlap significantly with those of Woodhouse *et al.* for all three cells (*p*<0.01, Fisher's exact tests) and the response to infection of genes identified in both studies is well conserved at 90-99% for each cell line.

By performing functional annotation clustering on the subset of 1181 genes found to be differentially expressed with the same response to infection over all infected versus control cell comparisons (figure 3, section G), we identifiy a core set of host cellular functions that are affected by HCV infection (supplementary table S2). Interestingly, the two most enriched clusters comprise genes involved in transcription. Zinc-finger domain containing proteins are highly over-represented in this set (249 genes are annotated with the SwissProt keyword “zinc-finger” [25]) (false-discovery-rate corrected p-value of 8.6×10^−29^). The change in expression of such a large number of zinc-finger domain encoding genes remains unexplained, particularly as many of these factors are not known to be associated with viral infection. However, of these 249 proteins, 143 are also annotated with the SwissProt keyword "transcription regulation". Given the scale of change in gene expression between infected and uninfected cells, extensive change in the expression of transcriptional regulators is fitting.

In addition, other cellular processes including microtubule organisation, ubiquitin and ubiquitin-like (ubl) conjugation pathway and DNA repair (particularly DEAD and DEAH box helicases) are enriched. HCV requires a functional microtubule network for entry into Huh-7.5 cells and early post-entry steps of infection through interaction with tubulin proteins [26]. We identify 11 tubulin isoforms that are either up- or down-regulated during HCV infection, indicating that HCV infection may exert control over the microtubule network at the level of transcription.

DEAD box helicases, RIG-I and IFIH1, are interferon stimulated genes (ISGs) that act to detect RNA viruses and initiate further interferon production [18]. Another DEAD box helicase, DDX3X, encodes a factor that is required for successful HCV replication [5], [27], [28]. However, DDX3X can also cause immune activation [29] and the role for this protein in HCV infection is unclear. RIG-I is transcriptionally upregulated in Huh-7 and Huh-7.5.1 cells but not Huh-7.5.1c2 following infection, and IFIH1 is transcriptionally upregulated in Huh-7.5.1 cells but not Huh-7 or Huh-7.5.1c2 following infection. These results indicate a potential weakness in the innate immunity of Huh-7.5.1c2 at the level of gene expression.

Virally triggered RIG-I mediated antiviral signaling evokes the production of type I interferon [30]. However, in our results we do not observe increase in transcription of type I interferon in either Huh-7 cells that have functional RIG-I or Huh-7.5 derived cells whose RIG-I gene has a known deactivating mutation [17]. This result suggests that HCV successfully attenuates interferon production. The virus can achieve interferon attenuation through several mechanisms including NS3/NS4A protease activity that disrupts both RIG-I and toll-receptor signaling [30]. The regulation of ISGs following infection of hepatoma cells was investigated. A list of ISGs was obtained from the ISG database [31] and a total of 455 genes were present in the ISG data and also present in our microarray gene set. We find that 62, 198 and 160 ISGs are differentially expressed in Huh-7, Huh-7.5.1 and Huh-7.5.1c2 cells, respectively, following HCV infection. However, these values do not represent statistically significant enrichment of ISGs, consistent with our observation regarding lack of significant transcriptional upregulation of interferon.

Ubiquitin conjugation has been identified as an important cellular function for both viral and bacterial pathogens [32]. Firstly, deubiquitylating enzymes (DUBs), such as ubiquitin specific peptidases (USPs) can modulate host cell innate immunity [32]. Ubiquitin-like protein ISG15 is an ISG that is expressed following infection and target proteins become “ISGylated” following conjugation of ISG15. Ubiquitin specific peptidase USP18 is involved in deISGylation to attenuate innate immunity [32], [33]. Another USP, USP7, is targeted by viral proteins. USP7 interacts with both the herpes-simplex virus protein ICP0 and Epstein-Barr nuclear antigen I and may have a role in regulation viral replication [34], [35]. We find that 27 USPs (though not including those specific USPs mentioned) are differentially expressed in one or more infected versus uninfected comparisons and, given all comparisons, there are 59 instances of differential expression of these genes from which 56 instances identify USP as transcriptionally upregulated in the infected cell.

Another DUB that has a role in pathogenic infection is CYLD. CYLD expression is induced in cells infected with *Haemophilus influenza* and the absence of this gene confers hypersensitivity to this bacterial pathogen [32]. In our results, CYLD is also upregulated in all HCV infected cells. Therefore it seems likely that DUB downregulation is a significant marker for HCV infection in these cells and this could be related to activation of an antiviral response, though presumably not via the RIG-I pathway or interferon upregulation. Interestingly, HCV NS5A has been shown by a yeast-two-hybrid assay to interact with USP19 [10], a DUB known to positively regulate cell proliferation [36]. The functional role of this protein interaction is not known and further experimental validation and investigation could provide valuable insight in to HCV infection.

Functional annotation clustering was repeated on gene sets from sections A, B, C and F of the Venn diagram in figure 3 (see supplementary table S2). The intersection of genes regulated with the same response from Huh-7.5.1 and Huh-7.5.1c2 but not Huh-7 infection studies (figure 3, section F, 1020 genes) is enriched for functions that can be directly attributed to heightened HCV infection, e.g., transforming growth factor β signaling [37] and a generally heightened metabolic state, e.g., positive regulators of transcription. Interestingly, one function whose enrichment is found in section C of the Venn diagram (corresponding to the set of genes differentially expressed following infection of Huh-7.5.1c2 cells), but not sections A or B (corresponding to infection of both Huh-7 and Huh-7.5.1), is apoptosis. More specifically, the most overrepresented annotation terms in this cluster refer to the negative regulation of cell death and these genes are predominantly upregulated in infected Huh-7.5.1c2. Specifically, there are 21 genes annotated with the GO term *negative regulation of cell death* and 16 of these are transcriptionally upregulated in infected Huh-7.5.1c2 cells. For example, NFKB and BCL2 genes are established as anti-apoptotic proliferative factors in human cancers and both are upregulated in infected Huh-7.5.1c2. Apoptosis is an important defense mechanism against infection that is initiated by the innate immune response [38] and this result indicates that Huh-7.5.1c2 could be a more permissive host for HCV than either Huh-7 or Huh-7.5.1 by being less prone to apoptosis.

### Subclones of Huh-7 derived cells have significantly altered gene expression

We performed gene expression analysis on six cell cultures that display a range of susceptibilities to HCV infection: HCV susceptible Huh-7, Huh-7.5.1 and Huh-7.5.1c2 and HCV resistant subclones of Huh-7.5.1, R1.09, R1.10 and R2.1. Differentially expressed genes were detected in all comparisons with a false discovery rate corrected p-value of <0.01 and minimum fold-change of 1.5. To identify differences in gene expression between these cells, ‘parent-child’ cell comparisons were made (see table 1 for a summary and supplementary S1 for full details).

**Table 1:** The number of differentially expressed (DE) genes identified in pairwise comparison between cells.

Original cell	Subclone cell	Total DE genes	Downregulated in subclone	Upregulated in subclone
Huh-7	Huh-7.5.1	2036	1148	888
Huh-7.5.1	Huh-7.5.1c2	187	119	68
Huh-7.5.1	R1.09	2830	1534	1296
Huh-7.5.1	R1.10	1714	771	943
Huh-7.5.1	R2.1	5682	2470	3212

The genes were found to be differentially expressed with a false discovery rate corrected p-value of <0.01.

**Figure 4:**
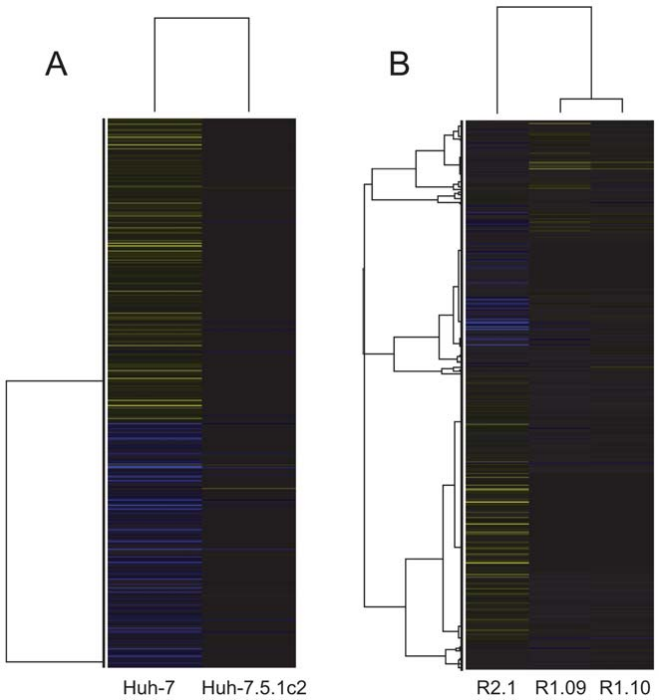
Hierarchical clustering plots showing the expression levels of differentially expressed genes between hepatoma cells. Genes are represented by horizontal bands and cells by columns. Bands are coloured blue if the gene is downregulated and yellow if they are upregulated relative to their expression in Huh-7.5.1. Greater colour intensity relates to a greater fold change. Black bands represent genes whose expression is a similar level to Huh-7.5.1. (A) Comparison of gene expression levels between susceptible cells. Here, the Huh-7.5.1c2 cell line is clearly more similar in gene expression to Huh-7.5.1 than Huh-7. (B) Comparison of gene expression levels between resistant cells and Huh-7.5.1. The R2.1 cell line is more divergent from Huh-7.5.1 than either R.109 or R1.10 in terms of gene expression. R1.09 and R1.10 show similar patterns of gene expression.

The pattern of gene regulation highlighted in heatmaps (figure 4) correlates with the subcloning of these cells, where the ‘child’ subclone retains a significant proportion of the gene expression profile of the ‘parent’. For example, many of the same genes are found to be differentially expressed with the same direction of regulation in the comparisons: (i) Huh-7.5.1 versus Huh-7 and Huh-7.5.1c2 versus Huh-7 (1479 genes in common) and (ii) R1.09 versus Huh-7.5.1 and R1.10 versus Huh-7.5.1 (1424 genes in common). Therefore, we represent cells and differential expression on a hierarchical tree structure figure 1. This shows all six cells and the total numbers of differentially expressed genes, both upregulated and downregulated. A full list of differentially expressed genes for each branch is given in supplementary table S3. A total of 7503 genes are differentially expressed following subcloning events. This represents a substantial proportion of both all genes on the microarray (42%) and the subset of those that are expressed in these cells (54%). This indicates that multiple changes in expression could contribute to the susceptibility to infection found among the hepatoma cells.

Genes that are differentially expressed following subcloning events were found to be enriched for specific biological annotations using functional annotation clustering. A brief summary of the most significant annotation clusters identified among each set of genes is given in table 2 and a full list of results is given in supplementary table S4. From table 2 it is clear that some areas of biological annotation are significantly enriched among more than one set of genes. For example, an annotation cluster corresponding to secreted glycoproteins and signal peptides appears in five out of the six sets and three out of five also include an annotation cluster that corresponds to proteins of the acute inflammatory response.

**Table 2:** Functional enrichment among significantly differentially expressed genes between original cells and subclones.

Original cell	Subclone cell	No. clusters	No. genes	Top 5 clusters		
		ES>2	ES>2	ES	No. genes	Annotations
Huh-7	Huh-7.5.1	15	866	5.81	565	Extracellular and secreted; signal peptide; glycoprotein; disulfide bond.
				4.93	119	Response to hormone stimulus and organic substance.
				3.92	47	Response to steroid hormone and glucocorticoid stimulus.
				3.52	48	Response to extracellular stimulus, nutrients, retinoic acid and vitamin A.
				3.37	37	Complement and coagulation cascades; acute inflammatory and defense response.
Huh-7.5.1	Huh-7.5.1c2	2	108	7.09	83	Glycoprotein; signal peptide; secreted; disulfide bond.
				4.93	74	Response to hormone stimulus and organic substance.
				10.55	416	Extracellular and secreted; signal peptide; glycoprotein; disulfide bond.
Huh-7.5.1	R1.1	18	704	5.03	88	Response to wounding; acute inflammatory and defense response.
				4.42	113	Mitosis; organelle fission; cell-cycle; M-phase.
				4.23	54	Enzyme inhibitor; endopeptidase and protease inhibitor; SERPIN family; reactive bond.
				3.60	50	Proteinaceous extracellular matrix; basement membrane.
				11.32	108	DNA replication and DNA metabolic process.
R1.1	R1.09	13	582	7.07	151	Mitosis; organelle fission; cell division; chromosome segregation;cell-cycle; M-phase.
				6.41	96	Chromosomal part; centromeric region; chromatin.
				5.46	140	DNA repair; response to DNA damage; stress response.
				4.85	45	Condensed chromosome; kinetochore; centromeric region .
R1.1	R1.10	2	108	4.12	55	Extracellular space.
				4.09	108	Secreted; signal peptide; glycoprotein; disulfide bond.
				6.34	165	Sequence-specific DNA binding; Homeobox DNA binding domain.
Huh-7.5.1	R2	25	1325	4.97	95	Embryonic morphogenesis; appendage development.
				4.93	40	Acute inflammatory response; acute phase.
				4.74	153	Acute inflammatory, wounding and defense response.
				4.59	73	Extracellular and secreted; signal peptide; glycoprotein; disulfide bond.

Shown are the number of functional annotation clusters that achieve an enrichment score (ES) of >2 and the number of differentially expressed genes that these clusters include. The right-most three columns give details of the top scoring annotation clusters (a maximum of 5 are shown).

**Figure 5:**
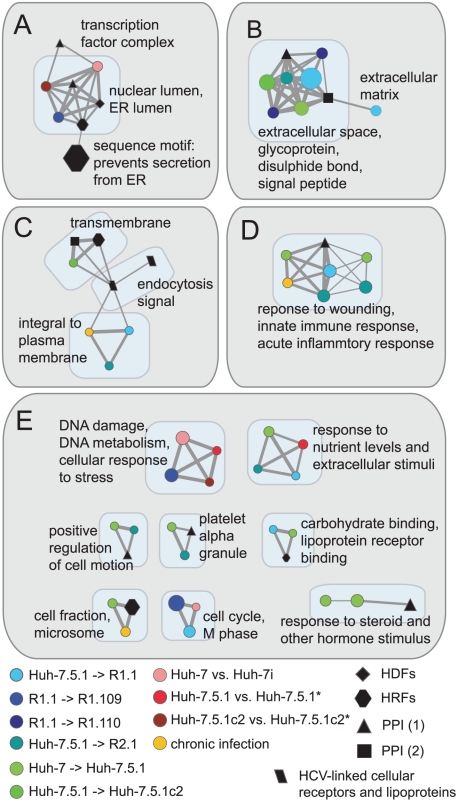
Functional annotation cluster networks from differentially expressed genes and other HCV-related data sources. These networks highlight areas of shared enriched function between gene sets that we identify as differentially expressed between hepatoma cells and also gene sets that relate to HCV infection. Nodes represent annotation clusters from the data source denoted by the node colour. Edges represent shared annotation terms between clusters. Only nodes that share at least 1/4 of annotating terms are connected by an edge. Node diameter is proportional to the level of enrichment of the biological function in the gene set. Edge width is proportional to the proportion of annotating terms shared between two clusters. Subnetworks A–D are those with >6 nodes, subnetworks shown in E have between 3 and 6 nodes. Annotation clusters from two PPI data sources are shown: PPI (1) from reference [10] and PPI (2) from reference [69].

To assess (i) overlap in biological function between each set of differentially expressed genes, (ii) overlap in biological function these gene sets may have with other genes that relate to HCV infection and (iii) to identify potential functions that contribute to susceptibility to HCV infection, we created a functional clustering network using all significant annotation clusters described in supplementary table S4. This functional clustering network comprises 36 subnetworks, shown in supplementary figure S1. These subnetworks correspond to areas of shared, enriched biological function between the gene sets. Figure 5 shows 12 of these subnetworks that include at least three nodes, at least one node corresponding to an enriched function from a subcloning event and at least one node corresponding to an enriched function from an additional data set linked directly to HCV infection. These visualisations highlight the possibility that changes in expression of genes of some of these particular functions may contribute to susceptibility to HCV infection in more than one subcloning event. For example, figure 5B shows that genes encoding protein products that interact with proteins of HCV and also genes that are differentially expressed between several independent subcloning events (corresponding to both increase and decrease in susceptibility to HCV infection), are all enriched for extracellular and secreted disulphide-bond containing proteins and signal peptides.

The subcloning of hepatoma cells has caused extensive changes to transcriptional activity, both in terms of the absolute number of differentially regulated genes and of biological functions affected. In the case of Huh-7.5 cells, RIG-I mutation is known to increase susceptibility to HCV infection and complementing these cells with wild-type RIG-I induces greater resistance [17]. However, differential expression of over 2000 genes from a variety of functions between Huh-7 and Huh-7.5.1 is not necessarily due to a single mutation of RIG-I, indeed this seems unlikely. Therefore, it is impossible to say whether Huh-7.5.1 derived cells are more susceptible than Huh-7 due to RIG-I alone, as change in regulation of other genes may also play a role. Huh-7.5 derived cells are commonly used for HCVcc but the extent to which subcloning-induced cellular alteration distances these cells from hepatocytes that are being modeled warrants greater consideration given the scale of change we report.

A previous study by Inoue *et al.* [39] made comparison of two Huh-7 subclones that had varying HCV replication efficiency. Inoue *et al.* identify 17 genes that have an increased level of expression and 19 genes that have a decreased level of expression in the more efficient of the cells. Though the present study and that of Inoue *et al.* have a shared aim, there is very little concurrence of results. This could be because Inoue *et al.* observed a different mechanism causing change in susceptibility to infection but it could also be due to the relatively small size of their result set. Regardless, our study has greater power as six Huh-7 derived subclones rather than two were analysed and expression of approximately 17 thousand genes, as opposed to approximately 8500 were assessed.

### Host factors linked to HCV are differentially expressed in subclones of Huh-7

#### HCV dependency factors

The 7503 genes differentially expressed following subcloning events are enriched for genes shown by siRNA gene knockdown to be necessary for HCV replication [5], [28], [40]–[42], termed HCV dependency factors (HDFs). A total of 292 genes that are expressed among the six cells are among HDFs and 176 of these genes are differentially expressed (*P* = 0.050, Fisher's exact test). This result indicates that differences in expression are likely to impact susceptibility of the cells to infection.

#### Cellular receptors and lipoproteins

**Figure 6:**
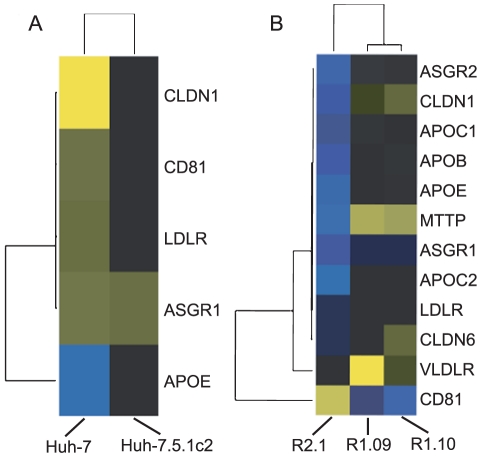
Hierarchical clustering plots showing the expression levels of differentially expressed HCV-linked cellular receptors and lipoproteins. Genes are represented by horizontal bands and cells by columns. Bands are coloured blue if the gene is downregulated and yellow if they are upregulated, relative to their expression in Huh-7.5.1. Greater colour intensity relates to a greater fold change. Black bands represent genes whose expression is a similar level to Huh-7.5.1. (A) Comparison of gene expression levels between susceptible cells. All genes shown are linked to HCV cell entry except for MTTP and APOB that are associated with release of HCV from the cell. The majority of these host factors that undergo a significant change in expression are found at a higher level in Huh-7 than either of the Huh-7.5.1 derived cells. (B) Comparison of gene expression levels between resistant cells and Huh-7.5.1. R1.09 and R1.10 cells have have a lower level of expression of CD81 than Huh-7.5.1. Though R2.1 cells have a relatively high level of CD81 expression relative to Huh-7.5.1, other cell entry factors are expressed at lower levels.

Cellular receptors and lipoproteins involved in HCV entry are differentially expressed in comparisons between both resistant and susceptible cells. Five genes – CLDN1, CD81, LDLR, ASGR1 and APOE – that promote virus entry are differentially expressed in a comparison between susceptible cells (figure 6). Of these five genes, all except APOE are downregulated in the Huh-7.5.1 cell, relative to Huh-7. APOE is transcriptionally upregulated in Huh-7.5.1 relative to Huh-7 (a fold-change 1.84) and ASGR1 is upregulated in Huh-7.5.1c2 relative to Huh-7.5.1 (a fold-change 1.57). Therefore, only APOE and ASGR1 are regulated in a manner that fits with the observed susceptibility to infection and neither undergo a substantial fold-change, thus, it does not seem likely that enhanced viral entry is a cause of the relative permissiveness to infection in Huh-7.5.1 and Huh-7.5.1c2 cells.

Ten factors that influence HCV entry are differentially expressed in comparison between resistant cells and the Huh-7.5.1 parent (DD81, ASGR1, ASGR2, CLDN1, CLDN6, VLDLR, LDLR, APOC1, APOC2 and APOE). CD81, an important coreceptor in HCV cell entry [13], is downregulated in both R1.09 and R1.10 relative to Huh-7.5.1. CD81 expression is significantly greater in R2.1 than Huh-7.5.1. However, eight of the nine remaining differentially expressed entry factors, all except for VLDLR, are downregulated in R2.1 relative to Huh-7.5.1, including four by more than 32-fold (APOC2, APOE, ASGR1 and CLDN1). These results are consistent with the findings of Zhong *et al.* who identify that low ectopic CD81 contributes to the resistance of the original R1 (but not R2) cell, from which R1.09 and R1.10 are descended. However, additional mechanisms of resistance must exist, as transduction of R1 cells to express CD81 did not fully restore the susceptibility to infection observed in Huh-7.5.1 [20]. From our expression analysis it does not appear that R1.09 and R1.10 cells lack other cell entry factors. Therefore, the mechanism of resistance to infection, additional to CD81-mediated entry in R1 derived subclones, is unlikely to be due to viral entry. Zhong *et al.* attribute infection resistance of R2 to processes other than CD81-mediated entry. However, we identify that many entry factors aside from CD81 are downregulated in R2.1. These factors include CLDN1, a component of tight junctions, the silencing of which prevents HCV entry into Huh-7.5 cells [43]. CLDN1 is transcriptionally downregualted in R2.1 compared to Huh-7.5.1, with a fold-change of approximately 36-fold, indicating impeded viral entry could contribute to R2.1 resistance to infection. However, like R1 and Huh-7.5 derived cells, evidence suggests that processes other than viral entry affect the permissiveness of R2.1 to infection by HCV; particularly as Zhong *et al.* show that HCV replicon replication was defective in R2 cells and over five thousand genes are differentially expressed following subcloning of R2.1 cell from Huh-7.5.1. Indeed, MTTP and APOB are associated with HCV particle formation and particle secretion [13]. Like the virus entry factors previously mentioned, both MTP and APOB are downregulated in R2.1 relative to Huh-7.5.1 by more than 32-fold. Therefore, it appears that R2.1 cells may also lack the ability to support aspects of the HCV replication cycle that take place post-entry.

#### Proteins associated with the HCV replicase complex

Host proteins from crude HCV replicase complexes were identified by mass spectrometry. These host proteins correspond to 236 host genes that we term host replication factors (HRFs) (supplementary table S5). A total of 212 HRFs were expressed among the cells that underwent microarray analysis and 145 of these are differentially expressed. This is significantly more than would be expected by random chance (*P* = 3.8×10^-5^, Fisher's exact test). This result suggests that the ability of these cells to support replication of viral RNA is unlikely to be consistent between these cells. Interestingly, among these 145 host genes are 12 (APOA1, APOE, CALR, CANX, FTH1, GNB2, HSPA5, OS9, PFN1, PPIB, SSR4, and TUBB2C) that encode a product known to interact with one or more HCV proteins [10]. For example, Chang *et al.* show that APOE is required for production of infectious HCV, probably for virion assembly rather than viral RNA replication [44] and CANX is involved in the folding of HCV glycoproteins [45].

Also, among the 145 host genes are two HDFs, DDOST and PPIA [5]. DDOST encodes a subunit (dolichyl-diphosphooligosaccharide-protein glycosyltransferase) of the oligosaccharyltransferase complex. DDOST is required during the late stages of HCV replication, possibly to perform an essential glycosylation step on HCV envelope proteins, E1 and E2 [5]. PPIA encodes a cyclophilin A the protein target of anti-HCV drug alisporivir [4]. Both DDOST and PPIA are downregulated in the R2.1 subclone compared to Huh-7.5.1 with fold change 2.16 and 1.82, respectively. APOE encodes apolipoprotein E, a constituent of lipoproteins. Surprisingly, the level of APOE gene expression in R2.1 cells is lower than in Huh-7.5.1 by over 100-fold. Taken together, these results suggest that the regulation of expression of DDOST, PPIA and particularly APOE might be sufficiently altered in R2.1 such that the cell are unable to form a competent HCV replication complex.

### Gene expression profiles highlight host factors and biological functions that are linked to HCV infection susceptibility

A significantly greater proportion of expressed genes appear on multiple branches of the tree of cell subclones (figure 1B) than would be expected by random chance (no Mann Whitney U test p-value exceeded 0.001 in 1000 permutations). This result indicates that the likelihood of undergoing a significant change in expression following subcloning is not equal for each gene. This may be due to a number of reasons including simple hyper- or hypo-variability of certain genes, or, more interestingly, some genes being differentially expressed multiple times during subcloning due to an effect they have on HCV infection susceptibility, their change in expression having been selected by subcloning.

To distinguish factors that may alter HCV susceptibility and to identify specific biological functions and proteins that may contribute to HCV infection susceptibility, we define a gene expression profile score that accounts for significant change in expression following independent subcloning events (see Materials and Methods). A negative score represents an antiviral expression pattern a positive score represents a proviral expression pattern. The frequencies of attained scores are given in table 3 and a full list of scores per gene is given in supplementary table S6. To demonstrate the significance of our measure, we tested whether other gene sets that are linked to HCV virus propagation have greater scores than would be expected. We find that HCV-linked cellular receptors and lipoproteins (including many factors involved with cell entry), genes that encode proteins that interact with HCV proteins and HRFs have a greater mean score than expected by random chance. The test result was not significant for HDFs (see table 4). These results indicate that our score is significant and is a useful measure for aiding identification of host cell factors that affect susceptibility to HCV infection. Furthermore, these results indicate that factors involved in virus entry into the cell, replication and those that have a direct association with proteins of HCV are likely to be important.

**Table 3:** The frequency of gene scores.

Score	Frequency total	Proportion
−5	1	0.013%
−4	11	0.15%
−3	77	1.03%
−2	592	7.89%
−1	3039	40.50%
0	1042	13.89%
1	2054	27.38%
2	554	7.38%
3	113	1.51%
4	19	0.053%
5	1	0.013%

Here, we give the profile scores, the frequency of the score among 7503 differentially expressed genes and the corresponding proportion. A negative score represents an expression profile that indicates a possible antiviral activity, whereas a positive score indicates a possible proviral activity.

**Table 4:** Mean profiles scores.

Gene set	No. of genes	Mean profile score	Permuted p-value
Receptors	14	0.5	0.0070
HRFs	173	0.283	<0.001
HDFs	230	−0.0174	0.173
HCV interacting	465	0.0452	0.0030

Details of the profile scores of four gene sets that we predict may have a higher score than would be expected by random chance. These gene sets are: HCV-linked cellular receptors and lipoproteins, the majority of which facilitate virus entry (but also particle formation and release, here labeled “receptors”), host factors that we isolate from vesicles that harbour the HCV replication complex (HRFs), host factors that are required for HCV replication determined by siRNA screen (HDFs) and HCV interacting proteins (HCV interacting). For each gene set we show the mean profile score and the significance of the score enrichment, determined by Mann Whitney U test permutation. For all gene sets other than HDFs, the profile scores are on average greater than we would expect by random chance, given a p-value cutoff of <0.05.

Though our score does penalise expression profiles that exhibit both antiviral and proviral tendencies, we would still expect biological functions that comprise genes that are hyper-variable in their expression in the hepatoma cell culture system to have a greater range of scores than biological functions whose genes tend to be expressed at a constant level. Therefore, we devised a test to ascertain whether the enriched functions that we highlight in figure 5 are simply hyper-variable or are consistent with a profile that corresponds to antiviral or proviral action (see Materials and Methods for details of this test).

We find that two areas of function are linked to the observed differences in susceptibility to HCV infection in more than one of the six cell cultures: (i) secreted signal peptides and glycoproteins (343 genes, figure 5B) and (ii) the acute and innate inflammatory responses (71 genes, figure 5D) were significant (*P* = 0.049 and *P* = 0.013 respectively). The innate immune response and particularly interferon-stimulated pathways play an important role in cellular defense against viral infection [46]. “Secreted signal peptides and glycoproteins” is a description relevant to proteins from a broad spectra of activities and there are several important factors among these that are differentially expressed which directly relate to HCV infection. These include TGF-β [47], low-density lipoprotein receptors and their associated proteins that have previously been discussed and TNF and serpin peptidase inhibitors [13], [48] (discussed in the next section).

We also define a set of ‘high-scorers’, genes with an absolute score ≥3. There are 222 high-scorers, representing approximately the top 3% of differentially expressed genes. Genes that scored >3 or <3 are listed in table 5. Among high-scorers are two HRFs, neutral cholesterol ester hydrolase 1 (NCEH1) and visinin-like 1 (VSNL1). NCEH1 catalyses hydrolysis of intracellular cholesterol ester, to produce free cholesterol. Free cholesterol may then be re-esterified or efflux to an extracellular cholesterol acceptor [49]. We identify NCEH1 as differentially expressed comparisons corresponding to: subcloning of Huh-7.5.1 from Huh-7, R1 from Huh-7.5.1, R1.09 from R1 and R2.1 from Huh-7.5.1. NCEH1 follows an antiviral expression profile without deviation and scores -4. Visnins are calcium sensor proteins that modulate multiple intracellular targets [50]. In contrast to NCEH, VSNL1 has a unanimously proviral expression profile of +3, as it is differentially expressed in three comparisons corresponding to: subcloning of R1 from Huh-7.5.1, R1.09 from R1, and R2.1 from Huh-7.5.1. Both of these genes are also differentially expressed in comparisons between infected and uninfected cells; NCEH1 is upregulated in both infected Huh-7.5.1 and Huh-7.5.1c2 cells compared to the uninfected cells, whereas VSNL1 is downregulated in Huh-7.5.1c2 cells following infection. Also, among high-scorers are three proviral HDFs: PROX1, GCAT and ATP10D. In agreement with their HDF status, these genes have unanimously proviral expression profiles, each scoring +3. These high-scoring genes that appear in multiple HCV-related data sources may have a significant role in HCV infection.

**Table 5:** Genes with top-scoring antiviral and proviral expression profiles.

Gene name	Profile score	infected vs. uninfected
trophoblast glycoprotein	−5	n n n
GalNAc-T7	−4	+ + +
sperm associated antigen 1	−4	+ + +
tubulin, alpha 1a	−4	n + +
neutral cholesterol ester hydrolase 1	−4	n + +
interleukin 17D	−4	n n –
proline-serine-threonine phosphatase interacting protein 2	−4	n + n
discoidin domain receptor tyrosine kinase 1	−4	n n +
lectin, galactoside-binding, soluble, 1	−4	n n +
ependymin related protein 1	−4	n n n
MHC class I polypeptide-related sequence B	−4	n n n
TIMP metallopeptidase inhibitor 1	−4	n n n
complement component 3	5	n – n
peptidoglycan recognition protein 2	4	– – –
potassium channel, subfamily T, member 2	4	– – –
coagulation factor XII (Hageman factor)	4	n – –
annexin A9	4	n – –
orosomucoid 2	4	n – –
complexin 1	4	n – –
hydroxysteroid (11-beta) dehydrogenase 2	4	n – –
transmembrane protein 86B	4	n – –
solute carrier family 7, member 10	4	n – –
haptoglobin	4	n – –
serpin peptidase inhibitor, clade C (antithrombin), member 1	4	n – –
haptoglobin-related protein	4	n – –
left-right determination factor 1	4	n – n
reelin	4	+ n n
argininosuccinate synthetase 1	4	n – n
ATP-binding cassette, sub-family B (MDR/TAP), member 4	4	n n n
KIAA1462	4	n n n
orosomucoid 1	4	n n n
coagulation factor V (proaccelerin, labile factor)	4	n n n

Genes with a profile score of <3 (antiviral profile) are listed above and >3 (proviral profile) are listed below the line. Also, given is the change in regulation of the gene in Huh-7, Huh-7.5.1 and Huh-7.5.1c2 cells (in that order) from uninfected versus infected comparisons, where “n” represents no significant differential expression, “+” represents upregulation in the infected cell and “–” represents downregulation in the infected cell.

### Investigation of HCV protein neighbourhoods reveals plausible mechanisms for change to infection susceptibility

**Figure 7:**
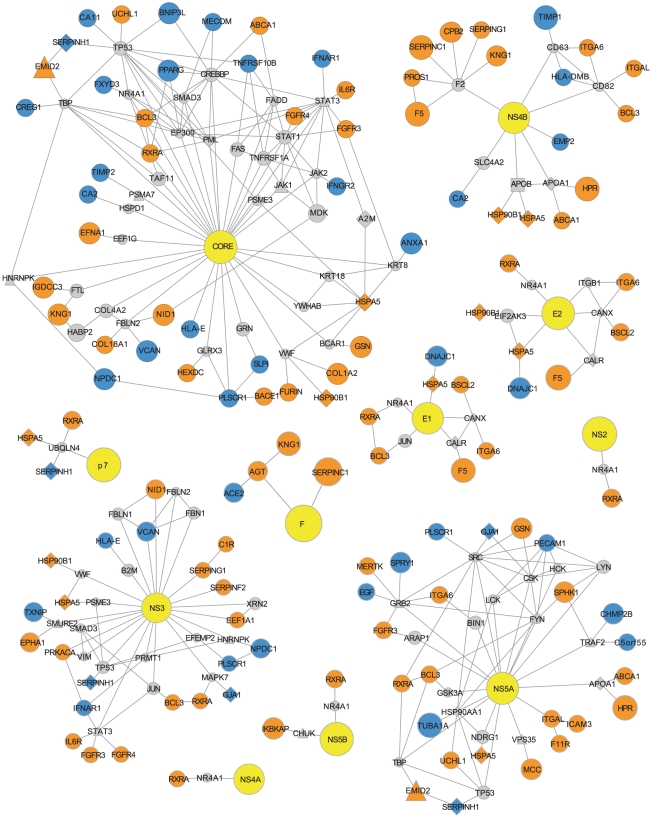
Protein interaction neighbourhoods of HCV proteins. HCV proteins are denoted by yellow nodes. Host proteins encoded by genes from either the high-scorer set or from significant antiviral and proviral biological functions are denoted by orange or blue nodes, indicating proviral or antiviral expression profiles, respectively. Other HCV interacting host proteins are denoted by grey nodes. Edges represent interactions between these proteins.

Investigation of the network neighbourhoods of HCV proteins could identify plausible mechanisms for change in HCV infection susceptibility (figure 7). In order to focus our search we only investigated differentially expressed genes from high-scorers or functional clustering networks corresponding to (i) secreted signal peptides and glycoproteins and (ii) the acute inflammatory response that we find to be significantly pro- and antiviral in their expression. In addition, we only evaluated interactions between these differentially expressed genes and HCV proteins, HDFs, HRFs and HCV-linked cellular receptors and lipoproteins.

#### Control over STAT3 protein activation

Stat3 (signal transducer and activator of transcription 3) is a transducer for a variety of signals including response to cytokines and growth factors. Upon activation by phosphorylation, Stat3 proteins dimerize and are translocated to the nucleus where they act as activators of transcription [51]. Stat3 is among the host cell interactants of HCV NS3 and core proteins [10] and is activated by core through a direct interaction, causing proliferation and possibly promoting tumorigenesis [52]. In addition, Stat3 has been shown in two independent siRNA screens to be essential for HCV replication [5], [28]. In contrast, other work has shown that Stat3 activation following interferon or IL6 treatment can prevent HCV subgenomic replicon replication by inducing an antiviral response [53]. Therefore, despite a clear importance, the effect of Stat3 signaling in HCV infected cells has yet to be fully understood.

In both the NS3 and CORE protein networks, we identify three host proteins, encoded by genes IL6R (interleukin 6 receptor), FGFR3 (fibroblast growth factor receptor 3) and IFNAR1 (interferon-α receptor 1), that act as activators of Stat3 activity. IL6R is an activator of Stat3, in response to interleukin 6 [51]. IL6 is included in gene sets taken from functional clustering networks (figures 5B and 0D) and has a profile score of +2. Specifically, IL6R is upregulated in Huh-7.5.1 in comparison with Huh-7 (fold change of 2.6) and downregulated in R2.1 in comparison with Huh-7.5.1 (fold change of 6.1). This change in regulation, combined with substantial fold changes purports a proviral action. However, this is not consistent with the findings of Zhu *et al.* [53], who show IL6 mediated signaling to be antiviral. FGFR3 is also included in the gene set taken from the functional clustering network (figure 5B), has a score of +2 and is downregulated in R2.1 (fold change of 1.6) and R1.10 (projected fold change of 1.8) following subcloning. Conversely, IFNAR1 has an negative score of -2, as it is downregulated in both the Huh-7.5.1 and R2.1 cells following subcloning, both with a fold change of 1.6. This activity is consistent with the findings of Zhu *et al.* on the basis that the IFN-induced antiviral activity can be mediated by this receptor.

#### Modulation of TNF-mediated signals and NF-kB activation

HCV modulates the host innate immune response using multiple strategies [54]. One of these strategies involves regulation of TNF-induced NF-kB, a transcriptional regulator and an important controller of inflammation and immune activation. Several HCV proteins are known to regulate NF-kB including NS5A, NS5B, core and F [55]–[58]. NF-kB activation is mediated through engagement of the TNF receptor. Upon stimulation, components of a signaling complex are recruited to the receptor. Signaling complex formation requires adaptor proteins including TRAF2 (TNF receptor associated factor family 2) [59]. NS5A appears to negatively regulate TNF-α-mediated activation of NF-kB through a direct interaction with TRAF2 [55]. However, TRAF2 has been shown by siRNA screen to be necessary for HCV replication [40], therefore it is unlikely that HCV infection simply requires suppression of TRAF2 activity. The sphingosine kinase 1 (SPHK1) and its product, an anti-apoptotic lipid mediator, sphingosine-1-phosphate, have recently been confirmed as important factors in TRAF2-mediated NF-kB activation [60]. The SPHK1 gene is expressed at a greater level in Huh-7.5.1 cells when compared to Huh-7 and resistant cells and is among the high-scorers with a score of +3, indicating that this gene may play an important proviral role in HCV infection, perhaps via a link to TRAF2 and an involvement in NF-kB induction, prevention of apoptosis and regulation of the innate immune response. We also identify two other genes among the subset of differentially expressed genes that we investigated that interact with TRAF2: CHMP2B (chromatin modifying protein 2B) and putative gene C5orf55. Both C5orf55 and CHMP2B have negative scores of −2 and −3, respectively, indicating a possible antiviral link. This provides additional evidence that TRAF2-related processes may have an effect during HCV infection.

#### Phospholipid scramblase 1 as an enhancer of interferon signaling

Interferons are important regulators of the innate immune response to viral infection [46]. Indeed, interferon-α is used as a treatment to reduce viral load in HCV infected patients [2]. PLSCR1 (phospholipid scramblase 1) is an interferon-stimulated gene that contributes to the interferon-mediated antiviral response. Though the underlying mechanism for antiviral activity of PLSCR1 remains to be fully understood, evidence indicates that this action is dually mediated at the cell membrane, where PLSCR1 can alter the distribution of phospholipids and in the nucleus, where this protein binds to DNA, possibly to potentiate transcription [61]. PLSCR1 appears in the gene set taken from the functional clustering network (figure 5D) and has a score of −2, as a result of being upregulated in all resistant cells (with fold changes of between 1.6 and 2.2), relative to Huh-7.5.1. In addition, PLSCR1 interacts with HCV core, indicating a mechanism through which HCV may act to control PLSCR1 signaling [10]. Therefore, PLSCR1 is a candidate for increased resistance to infection of R1.09, R1.10 and R2.1 cells.

#### Cholesterol efflux

A link between cholesterol efflux and HCV infection has been made previously. The scavenger receptor SR-BI mediates cellular uptake of cholesterol and the flux of cholesterol between HDL and the cell [62]. SR-BI is also an important HCV virus entry factor, possibly promoting viral entry through regulation of plasma membrane organisation, being a provider of cholesterol and interaction with other entry factors [13]. Virion-associated cholesterol is also a requirement of HCV infectivity [63]. We have previously mentioned the antiviral expression profile of NCEH1 and it’s inclusion among high-scorers and HRFs. Another host protein with a related role is ATP-binding cassette protein (ABCA1). This protein is a cholesterol efflux pump for removal of cellular lipids [64]. ABCA1 is among the gene set taken from the functional clustering network (figure 5B) and unlike NCEH1 it has a proviral profile score of +2. The ABCA1 encoded protein effluxes cholesterol to apolipoprotein A-I, a major constituent of HDL and these two proteins interact directly. Apolipoprotein A-I is present among HRFs and also interacts directly with HCV NS5A, probably as part of HCV-associated lipid metabolism dysregulation [65]. This evidence suggests that enzymes with the ability to alter the balance of cholesterol efflux may also impact HCV infection. Therefore, ABCA1 and NCEH1 may have an effect on susceptibility to HCV infection in hepatoma cells.

#### Serpins as mediators of HCV NS3 protein activity

HCV NS3 protein is a serine protease that contains a helicase domain and a serine protease domain. NS3 is responsible for cleavage of viral polyproteins and disruption of host innate immune response [66]. NS3 protease action is inhibited by serpin C1 through a direct physical interaction [48] and serpin-mediated inhibition of NS3 has been proposed as a possible anti-HCV therapy [48], [67]. However, we find that SERPINC1, the gene that encodes serpin C1 and a second serpin encoding gene SERPINA6 are among the high scorers with proviral expression profiles that score +4 and +3 respectively and these instances of differential expression also include substantial fold-changes. Serpin C1 has also been found to interact with the HCV F protein [68], NS3 also interacts with other serpins G1 and F2, and serpins C1 and G1 are both found in the NS4B PPI network neighbourhood (figure 7). The latter serpin genes all have proviral expression patterns. Furthermore, we also identified that serpins encoded by genes SERPINH1 and SERPINA1 are part of the HCV replication complex. These results raise the question of whether serpins play an additional proviral role in mediation of NS3 (and possibly F and NS4B) protein activity HCV life cycle, possibly as part of the HCV replication complex.

### Conclusion

In this study we performed multiple genome scale expression studies of Huh-7 derived hepatoma cells with the aim of identifying genes and biological functions that have a significant role in HCV infection. This permitted a detailed account of changes to gene expression caused by HCV infection, determined key differences between cells commonly used for HCVcc and implicated novel host factors in determining cellular permissiveness to infection.

Firstly, by comparing uninfected and infected hepatoma cells we identified a set of host cellular functions that are regulated during HCV infection including proteins associated with microtubule organisation, ubl conjugation, zinc-finger domain-containing transcription factors and proteins with helicase activity (supplementary table S2). Though proteins involved in microtubule organisation, ubiquitination and DEAD-box helicases have previously been identified as differentially expressed following HCV infection of hepatoma cells *in vitro* [12], the sensitivity of our study has highlighted the breadth of regulation of genes with these functions. In addition, we identify transcriptional upregulation of ubiquitin specific peptidases as a particular mark of HCV infection in Huh-7 derived cells. Furthermore, our results indicate that transcriptional upregulation of anti-apoptotic and proliferation stimulating factors may be a cause of increased permissiveness to HCV infection in Huh-7.5.1c2 cells.

Secondly, we examined the expression profiles of six hepatoma cells that have been subcloned from Huh-7, including three cell types that are resistant to HCV infection and genes differentially expressed between subcloned cells and their parent cells were identified. We were able to confirm that cells derived from the R1 subclone have significantly reduced levels of CD81 owing to a mechanism that acts at the level of gene expression. Additionally, we identified 236 host factors that are associated with the HCV replication complex in the membranous web of infected cells (HRFs, supplementary table S5). This is the largest set of HRFs that has been identified to date. From HRFs we implicate change in expression of APOE, DDOST and PPIA in the resistance to infection of the R2.1 cell. We also identify a subset of HRFs that interact with HCV proteins including APOE, CALN that are known to be involved in production of HCV [44], [45]. We scored genes according to their expression profile and used these scores to identify antiviral and proviral candidate genes. Table 5 lists the top scoring genes that include both novel candidate host factors and factors linked to HCV replication, such as tubulin-α [26] and two HRFs, NCEH1 and VSNL1; NCEH1 is a potentially antiviral factor and VSNL1 is a potentially proviral factor.

Our analysis of HCV infected cells also highlighted the ability of HCV to attenuate interferon upregulation, even in the Huh-7 cell that, unlike Huh-7.5 derived cells, is not reported to have a defective RIG-I signaling pathway. However, when we performed a meta-analysis of gene expression in the six uninfected Huh-7 derived cells, five of which are subcloned from Huh-7.5, we identify that the acute and innate inflammatory responses, as well secreted signal peptides and glycoproteins, are likely to be linked to differences in susceptibility to infection between these subclones. Furthermore, we can predict that these mechanisms of susceptibility or resistance to infection are independent of RIG-I signaling. Following these observations network neighbourhoods of HCV proteins were explored and hypotheses for changes to susceptibility to infection were postulated that involve novel HCV-related factors including ABCA1, SPHK1 and CHMP2B, in addition to supporting previously implicated factors such as PLSCR1 [10] and STAT3 [10], [52].

Interestingly, we find that secreted proteins (particularly glycoproteins) are linked to HCV infection. Genes with this annotation are over-represented among differentially expressed genes from multiple parent-child subclone comparisons and we identify these as having more significant proviral and antiviral expression profiles than would be expected than by random chance. Among these secreted proteins are factors involved in coagulation, such as complement components, serpins and coagulation factors and these factors have largely proviral expression profiles (see table 5 for some examples). Indeed, members of complement and coagulation pathways have previously been identified as potentially important cellular cofactors of NS4B through yeast two-hybrid and functional network analysis [69]. Also, among HRFs are factors that have a role in folding and secretion of coagulation factors, such as CALR and CANX [70], and CANX is also involved in production of HCV glycoproteins [45]. Hence, changes in HCV infection susceptibility could relate to the ability of the cells to produce viral glycoproteins. For example, HSPA5 encodes a heat-shock protein that is involved in protein folding and assembly in the endoplasmic reticulum [71]. HSPA5 is downregulated in all HCV resistant cell types compared to Huh-7.5.1 with approximate fold-changes between 1.5 and 2, though with highly significant probability (fdr <1×10^-7^ in each case). Other host factors with chaperone and protein folding activity that achieve high profile scores and appear in HCV protein network neighbourhoods include heat-shock proteins DNAJC1 and HSP90B1 [72], [73]. Another heat-shock protein, Hsp90, has been shown previously to form a complex that includes HCV NS5A and has an important role in HCV RNA replication [74]. Investigation of the ability of these chaperones to influence virus protein production will potentially identify additional mechanisms important to HCV infection.

Overall, our study builds upon current knowledge of infection and our results may contribute to the development of new antiviral treatments to counter the global HCV problem, particularly where we identify potentially proviral proteins that could act as drug targets. Further study, such as genomic sequencing of Huh-7 derived cells would provide greater insight in to the extent that these cells mutate in order to effect the extensive differences in gene expression that we observe following subcloning. Changes to susceptibility to infection could then be attributed to gene-specific mutations, in the same way that Huh-7.5 susceptibility has been linked to mutation of RIG-I. Genome sequencing may also highlight other previously undetected mutations in Huh-7.5.1 as well as other Huh-7 derived cells to further our understanding of HCVcc systems and their suitability for modeling infection.

## Materials and Methods

### HCV-resistant cells R1.09, R1.10 and R2.1

R1 and R2 cells were obtained from stocks held at the The Scripps Research Institute, La Jolla, California, from a previous study [20]. Cryopreserved cells were thawed and put in culture. Initially, both cells displayed very limited viability after two independent thawing attempts of the original cryopreserved stock. Nevertheless, we were able to rescue both cell lines by slowly expanding the surviving colonies, subsequently labelled R1.1 and R2.1. To verify the resistance of these cell lines to HCV Con1 (genotype 1b) subgenomic (SG)-replicon replication, R1.1, R2.1 and the parental Huh-7.5.1 cells were transfected with the corresponding replicon RNA encoding a neomycin resistance gene and the formation of G418 resistant cell clones was monitored. R1.1 and R2.1 cells are partially resistant to HCV replication (supplementary figure S2A). However, a higher percentage of cell clones in the R1.1 and R2.1 cell lines seem to support HCV replication than was previously observed in R1 and R2 cells before cryopreservation [20]. To verify that this result was reproducible for other replicon RNA preparations, we repeated the transfection experiment using Con1 full-length replicon RNA to select HCV replication resistant cell clones within the R1.1 and R2.1 cell populations (supplementary figure S2B). R1.1 and R2.1 cells were subcloned by limiting dilution on feeder cells. Individual subclones were tested for resistance to subgenomic Con1 replicon replication and subclones R1.09, R1.10 and original R2.1 cells were selected for further analysis (supplementary figure S2C). R1.09, R1.10, R2.1 and Huh-7.5.1 were harvested for microarray analysis.

### HCV susceptible cells, Huh-7, Huh-7.5.1 and Huh-7.5.1c2

Three Huh-7 derived cell cultures that can support HCV infection [75], [76] were used in this study – Huh-7, Huh-7.5.1 and Huh-7.5.1c2. Cells were obtained from stocks held at the The Scripps Research Institute, La Jolla, California. All cells have been used in previous studies (Huh-7 [76], Huh-7.5.1 [16] and Huh-7.5.1c2 [19]).

The HCV susceptible cell types, Huh-7, Huh-7.5.1 and Huh-7.5.1c2, were infected with wild type JFH-1 (genotype 2a) virus at an moi = 0.05. Infected cells were harvested for microarray analysis when virtually 100% of the cells were infected as determined by staining of cells for viral E2 protein at 3 (Huh-7.5.1c2), 4 (Huh-7.5.1) and 7 (Huh-7) days post inoculation (supplementary figure S2D). Uninfected controls were harvested at the same time point as infected cells. The infectivity of supernatant produced from infected cells was measured at 2, 4 and 8 days post infection (supplementary figure S2E). These results show that Huh-7 has the lowest susceptibility and Huh-7.5.1c2 the greatest susceptibility to HCV infection. In addition, uninfected Huh-7, Huh-7.5.1 and Huh-7.5.1c2 cells were harvested at 20 hours post infection in order to perform direct comparisons of their gene expression profiles by microarray analysis.

### Preparation of microarrays

Cell cultures were centrifuged at 1000 rpm for 5 minutes and the cell pellet was resuspended in 350 *µ*l lysis buffer (Qiagen). Each lysate was homogenised with a Qiashredder column (Qiagen) and the RNA was extracted using the RNeasy Mini Kit following the manufacturers instructions. On-column DNA digestion was carried out by means of the RNase-Free DNase Set (Qiagen) and the integrity of the RNA was confirmed via Agilent (RIN of 9.7–10). 100 ng of RNA from each sample was used to prepare cDNA with the Affymetrix GeneChip 3 IVT Express Kit and hybridised to Affymetrix U133 Plus 2 microarrays following the manufacturer's instructions.

The washing and staining procedure was performed in the Affymetrix Fluidics Station 450. The probe array was exposed to 10 washes in 6×SSPE-T at 250 C followed by 4 washes in 0.5×SSPE-T at 500 C. The biotinylated cRNA was stained with a streptavidin-phycoerythrin conjugate, final concentration 2 mg/ml (Molecular Probes, Eugene, OR) in 6×SSPE-T for 30 min at 250 C followed by 10 washes in 6×SSPE-T at 250 C An antibody amplification step followed using normal goat IgG as blocking reagent, final concentration 0.1 mg/ml (Sigma) and biotinylated anti-streptavidin antibody (goat), final concentration 3 mg/ml (Vector Laboratories). This was followed by a staining step with a streptavidin-phycoerythrin conjugate, final concentration 2 mg/ml (Molecular Probes, Eugene, OR) in 6×SSPE-T for 30 min at 250 C and 10 washes in 6×SSPE-T at 250 C. The probe arrays were scanned at 560 nm using a confocal laser-scanning microscope (Affymetrix Scanner 3000 7G). CEL files were generated and used for further analysis. All microarray procedures were done at AROS, Denmark.

### Computational analysis of microarray probe set intensity data

All analysis of microarray intensity data was carried out using R statistical software [77] including Bioconductor [78]. GeneChip® probe sets definitions were assigned using Entrez gene version 12.1 of a custom chip description file (CDF) from Psychiatry/MBNI Microarray Lab [79]–[82]. This CDF included 17726 probe sets that correspond to an NCBI gene.

Initial quality checks of each chip were carried out using Bioconductor core tools and package affyQCReport [83]. Quality checks and visual inspection of array intensities showed that the quality of the array data was acceptable. Robust Multichip Average (RMA) expression values [82], [84], [85] were computed using the Bioconductor affy package. Genes that were called “not present” on all 39 GeneChip array data sets using Microarray Suite version 5.0 (MAS5) presence calls were removed [86], leaving a total of 13760 genes (supplementary table S7). This set was used throughout as a background for statistical tests and will be referred to as the microarray gene set.

Exploration of RMA expression values across all microarray chips was performed by PCA using singular value decomposition (SVD). PCA was carried out using the Bioconductor package pcaMethods [87] and PCA results were plotted using the R package scatterplot3d. PCA results (supplementary figure S3) show that biological replicates cluster together. The greatest variation is seen between replicates from experiment 1 that have been in culture for a longer time period with no JFH-1 infection. However, replicates that have been infected with JFH-1 cluster very closely, indicating a clear gene expression response to infection. Replicates from experiment 2 are all very tightly clustered and Huh-7.5.1 replicates from experiment 2 generally cluster with other uninfected Huh-7.5.1 derived cells. The PCA analysis results showed no major outliers or unexpected results and the array quality was shown to be acceptable. Therefore, the microarray data appeared sufficiently reliable to conduct analysis to identify differentially expressed genes.

Probability, false discovery rate (FDR) [88] and fold-change values for differential expression of genes between cells, using three biological replicates from each, were calculated in a pairwise manner using the limma method [89]. Genes were defined to be significantly differentially expressed if they achieved an FDR of <0.01 and a minimum fold-change of 1.5. Hierarchical clustering of genes across cells was performed in R using Pearson's r correlation (genes) and Spearman's rank correlation (cells) and results were visualised using the gplots package [90].

The design of our experiment permitted HCV infected cells Huh-7, Huh-7.5.1 and Huh-7.5.1c2 to be compared to uninfected controls harvested after 20 hours in culture and also uninfected controls harvested at the same time point as infected cells. We define those genes that are differentially expressed due to infection as genes that are significantly differentially expressed in the same direction (up- or downregulated) over both comparisons, as this will filter out genes whose expression fluctuates due to additional time spent in culture. We take the p-value, fdr and fold-change values from the comparison with the most conservative comparison.

MIAME compliant raw and processed gene expression data is available for download as a GEOarchive [91], accession number GSE29889.

### Purification of crude replicase complexes (CRCs) for proteomic analysis by mass spectrometry

CRC preparations were produced using a protocol described by Quinkert *et al.* [92]. Protein samples from purified, proteinase K-treated CRCs were fractionated by one-dimensional SDS-PAGE and the gel was segmented according to molecular weight. Proteins contained in gel segments were digested with proteinase (trypsin/chymoptrypsin) prior to analysis by liquid chromatograph-mass spectrometry (WFZ Fungene, Greifswald). Using this method, 236 host-encoded proteins were identified as components of HCV replication complexes.

### Collection of external gene sets

Four sets of genes that relate to replication of HCV were collected from external sources:

- Genes that encode products that interact with proteins of HCV were retrieved from two studies [10], [69]. We identified 465 such genes that correspond to an NCBI protein.
- A non-redundant list of genes that have been identified by siRNA screen to play a significant role in HCV replication were obtained from five separate studies [5], [28], [40]–[42]. This list contains 399 genes, including 363 that were shown to be necessary for propagation of HCV (proviral) in one or more study, and 37 that have been shown to be detrimental to HCV propogation (antiviral) from the study by Brass *et al.*. Brass *et al.* identify 203 genes (193 proviral, 10 antiviral) that act during an early stage of infection and 59 genes (44 proviral, 15 antiviral) that act during a late stage in the viral life cycle.
- Human host genes that are differentially regulated due to chronic infection by HCV were mapped from an *in vivo* study comparing chronically infected chimpanzees to uninfected controls [93].
- 27 Cellular receptors and lipoproteins that are thought to have a role, either via positive or negative, in regulating HCV virion cell entry or particle release were manually curated from a recent review article [13]. These genes, by gene symbol are CD81, CD209, CLEC4M, CLDN1, CLDN6, CLDN9, SCARB1, LDLR, VLDLR, ASGR1, ASGR2, OCLN, APOC1, APOC2, APOE, APOB, MTTP, ISGF8, SAAL1, SAA4, EIF2A, EIF2AK2, IFNA2, IFNA5, IFNA8, IFNA16 and APOC3.

### Assignment of differentially expressed genes to a cell tree and calculation of expression profile scores

A tree showing the lineage of relevant cells is shown in figure 1. Changes in gene expression were assigned to branches of this tree directly from comparison of the ancestor and descendent cell, except for those branches linking R1 to other cells, which were imputed using a simple parsimony method. Genes identified as differentially expressed in comparisons Huh-7.5.1 versus R1.09, Huh-7.5.1 versus R1.10 or R1.09 versus R1.10 were assigned to: (i) The branch linking Huh-7.5.1 and R1 if the gene is called differentially expressed and undergoes the same direction of regulation (up/down) in both comparisons involving Huh-7.5.1. (ii) The branch linking R1 to a descendent cell if the gene is only called differentially expressed with a given direction in just one of the two comparisons involving Huh-7.5.1. (iii) The branch linking R1 to a descendent cell if the gene is called differentially expressed in the comparison between R1.09 and R1.10; in this case, the descendent and direction of regulation is chosen from the comparison involving Huh-7.5.1 and a descendent where the largest fold change is observed.

To test whether genes appear more regularly on multiple branches of this tree than would be expected by random chance a permutation test was used. In a single permutation of this test we assign genes to branches of a model tree by randomly selecting them from the microarray gene set, selecting the same number of genes per branch as observed in the real tree. We then derive the frequency distribution for genes appearing in branches of the model tree. The frequency distribution for the model data is compared to the frequency distribution from the real data by Mann-Whitney U test to generate a p-value, testing the hypothesis that the real data values will be greater than that of the model data.

Using the tree and associated sets of differentially expressed genes, expression profiles consisting of an integer score per gene were derived. Where a gene changes regulation on a branch linking a more HCV susceptible parent cell to a more HCV resistant descendant cell, the profile scores -1 if the gene is upregulated (antiviral) and +1 if the gene is downregulated (proviral). Where a gene changes regulation on a branch linking a more resistant parent to a more susceptible descendant, the profile scores -1 if the gene is upregulated and +1 if the gene is downregulated. The overall gene profile score is calculated as the sum of these values over all tree branches. Genes that are present in the microarray gene set but not among the differentially expressed genes on this tree were assigned a score of zero.

A permutation test was used to test the hypothesis that a given gene set comprises genes with greater expression profile scores than would be expected by random chance. A distribution of scores from a set of subject genes were compared against the distribution of scores from all remaining genes from the microarray universe in a one-tailed Mann Whitney U test to identify whether the subject set has significantly greater scores than expected. The test statistic (U) for this test was recorded. We then repeated this test using a subject gene set of randomly selected genes, recording U for every permutation. 1000 permutations were carried out. The p-value was determined to be the proportion of times a more significant U value was generated by random permutation than for a real subject gene set.

A permutation test was used to test if genes from a given set have greater absolute expression profile scores given the number of times that the genes are differentially expressed, than would be expected by random chance. Using this measure we can ascertain whether the subject genes have a genuine tendency to be proviral or antiviral, or if they are simply hyper-variable in their gene expression. For each gene in the set we calculated a normalised score (*S*-norm), being the expression profile score divided by differential expression count. If the gene set has a significant tendency for the genes to be proviral or antiviral rather than hyper-variable, we would expect the *S* -norm to be greater than those for randomly selected genes. Hence, we test whether the genes of the subject set have a greater mean *S* -norm than a gene set of the same size, selected at random from the microarray gene universe. When randomly sampling genes, we maintain the distribution of differential expression counts observed in the subject gene set using rejection sampling. The p-value for the test was determined to be the total number of times that the random set has a greater mean average *S* -norm than the subject set, divided by the number of permutations. 1000 permutations were performed.

### Analysis of the biological function of genes

Gene sets were subjected to functional enrichment using the Database for Annotation, Visualisation and Integrated Discovery (DAVID) version 6.7 functional annotation clustering and functional annotation chart tools [94], [95]. In both cases a custom background population consisting of the microarray gene set was used. All remaining DAVID 6.7 tools settings were left as the default. The set of differentially expressed genes from the comparison between Huh-7.5.1 and R2 cells was limited to 3000 by selecting the set with lowest p-values for differential expression, as this corresponds to the maximum gene set size that can be analysed using DAVID. Annotation clusters were deemed to be significant if the enrichment score was >2, this corresponds to a geometric mean from all term enrichment p-values of 0.01.

Functional clustering networks were produced using results from DAVID functional annotation clustering. Significant annotation clusters were represented as nodes, where the node diameter is proportional to the enrichment score. Edges signifying shared annotation were created between nodes where the annotation clusters being represented share at least one quarter of annotating terms, where the edge diameter is proportional to the fraction of shared annotation terms. Networks were visualised using Cytoscape [96].

### Construction of HCV protein network neighbourhoods

Human protein interaction data was retrieved from multiple sources compiled by the National Centre for Biotechnology Information (NCBI) and available as a download (ftp://anonymous@ftp.ncbi.nlm.nih.gov/gene/GeneRIF/interactions.gz). NCBI interactions data was downloaded on 10th August 2010. Physical protein-protein binding interactions were taken per gene, not including homo-dimer interactions (i.e., self edges in the network). All retrieved interactions, were used to compile a global interaction network where each interaction is treated uniformly, consisting of 48467 interactions between 10360 genes. HCV-human PPI data was retrieved from two HCV-human interaction studies [10], [69], on a per HCV protein-human gene basis and consisted of 533 interactions including 465 human genes and 11 HCV proteins. HCV protein network neighbourhoods were constructed that included specific differentially expressed genes and data from additional data sets. First a node corresponding to a HCV protein was created, next additional nodes corresponding to HCV-interacting host factors were added and finally we added nodes corresponding to differentially expressed genes that share an interaction with factors already present in the network. Finally, nodes that correspond to those non-differentially expressed host genes that do not share an interaction with a differentially expressed gene were pruned. The result is a network where the maximum path length from the HCV protein is two and the maximum path length for a non-differentially expressed host gene is one. Networks were visualised using Cytoscape [96].

## Supporting Information

Figure S1 Functional annotation cluster networks from differentially expressed genes and other HCV-related data sources. These networks highlight areas of shared enriched function between gene sets that we identify as differentially expressed between hepatoma cells and also gene sets that relate to HCV infection. Nodes represent annotation clusters from the data source denoted by the node colour. Edges represent shared annotation terms between clusters. Only nodes that share at least 1/4 of annotating terms are connected by an edge. Node diameter is proportional to the level of enrichment of the biological function in the gene set. Edge width is proportional to the proportion of annotating terms shared between two clusters.
(PDF)

Figure S2 (A) Huh-7.5.1, R1.1 and R2.1 G418 resistant colonies transfected with HCV genotype 1b (Con1) subgenomic replicon encoding a neomycin resistance gene. (B) Huh-7.5.1, R1.1 and R2.1 G418 resistant colonies transfected with Con1 full-length replicon RNA encoding a neomycin resistance gene. (C) Huh-7.5.1, R1.09 and R1.10 G418 resistant colonies transfected with HCV genotype 1b (Con1) subgenomic replicon encoding a neomycin resistance gene. (D) Staining of HCV infected cells for viral E2 protein at 3 (Huh-7.5.1c2), 4 (Huh-7.5.1) and 7 (Huh-7) days post inoculation.(E) Infectivity of supernatant produced from infected cells at 2, 4 and 8 days post infection.
(PDF)

Figure S3 PCA analysis was carried out on RMA expression values of each array. Principal components 1, 2 and are plotted for each array.
(PDF)

Table S1 Results of differential expression analysis including Entrez gene ID, cell comparison in which the gene is differentially expressed, log fold change, p-value and corrected p-value.
(TXT)

Table S2 Output from DAVID 6.7 functional annotation clustering on subsets of genes that are differentially expressed following HCV infection. Only annotation clusters that have an enrichment score _>2_ are shown. Sheets A–G correspond to the gene sets that are illustrated in Figure 3 in the main text.
(XLS)

Table S3 Differentially expressed genes assigned to branches of the tree of cells. Shown are the Entrez gene IDs, the tree branch to which the differentially expressed gene is ascribed and the cells in which the gene is up- and downregulated.
(TXT)

Table S4 Output from DAVID 6.7 functional annotation clustering. The first six sheets show results for gene sets that are differentially expressed on a specific branch of the tree of cells. The following three sheets show results from genes that are differentially expressed in HCV susceptible cells following infection. The remaining six sheets show results for other HCV-related data sets: HCV-linked cell receptors and lipoproteins, HRFs, HDFs, two sets of HCV-protein interacting factors (from studies [10] and [69], respectively) and genes differentially expressed during chronic HCV infection [93]. Only annotation clusters that have an enrichment score _>2_ are shown. Each sheet shows results from a different branch and sheets are named accordingly.
(XLS)

Table S5 List of HCV replication factors (HRFs). Shown is the Enrez gene ID, gene name and protein accession.
(XLS)

Table S6 Expression profile scores for genes that were differentially expressed (DE) and assigned to a tree branch (supplementary table S5.6). Shown are the Entrez gene ID, number of times the gene is found to be differentially expressed, the overall pattern of gene regulation, the score (s) and normalised score (s-norm).
(TXT)

Table S7 The microarray gene set following removal of genes that are not expressed in any cell type (see Materials and Methods for details). Shown are the array annotation ID, Entrex gene ID, gene symbol and gene name.
(TXT)
