# Supplementary material for: An Integrated Transcriptomic and Meta-Analysis of Hepatoma Cells Reveals Factors That Influence Susceptibility to HCV Infection
Source: PLoS One. 2011 Oct 25;6(10):e25584. doi: 10.1371/journal.pone.0025584 (PMC3201949; doi:10.1371/journal.pone.0025584)
Supplement: Figure S1 — Functional annotation cluster networks from differentially expressed genes and other HCV-related data sources. These networks highlight areas of shared enriched function between gene sets that we identify as differentially expressed between hepatoma cells and also gene sets that relate to HCV infection. Nodes represent annotation clusters from the data source denoted by the node colour. Edges represent shared annotation terms between clusters. Only nodes that share at least 1/4 of annotating terms are connected by an edge. Node diameter is proportional to the level of enrichment of the biological function in the gene set. Edge width is proportional to the proportion of annotating terms shared between two clusters. (PDF) [file pone.0025584.s001.pdf]

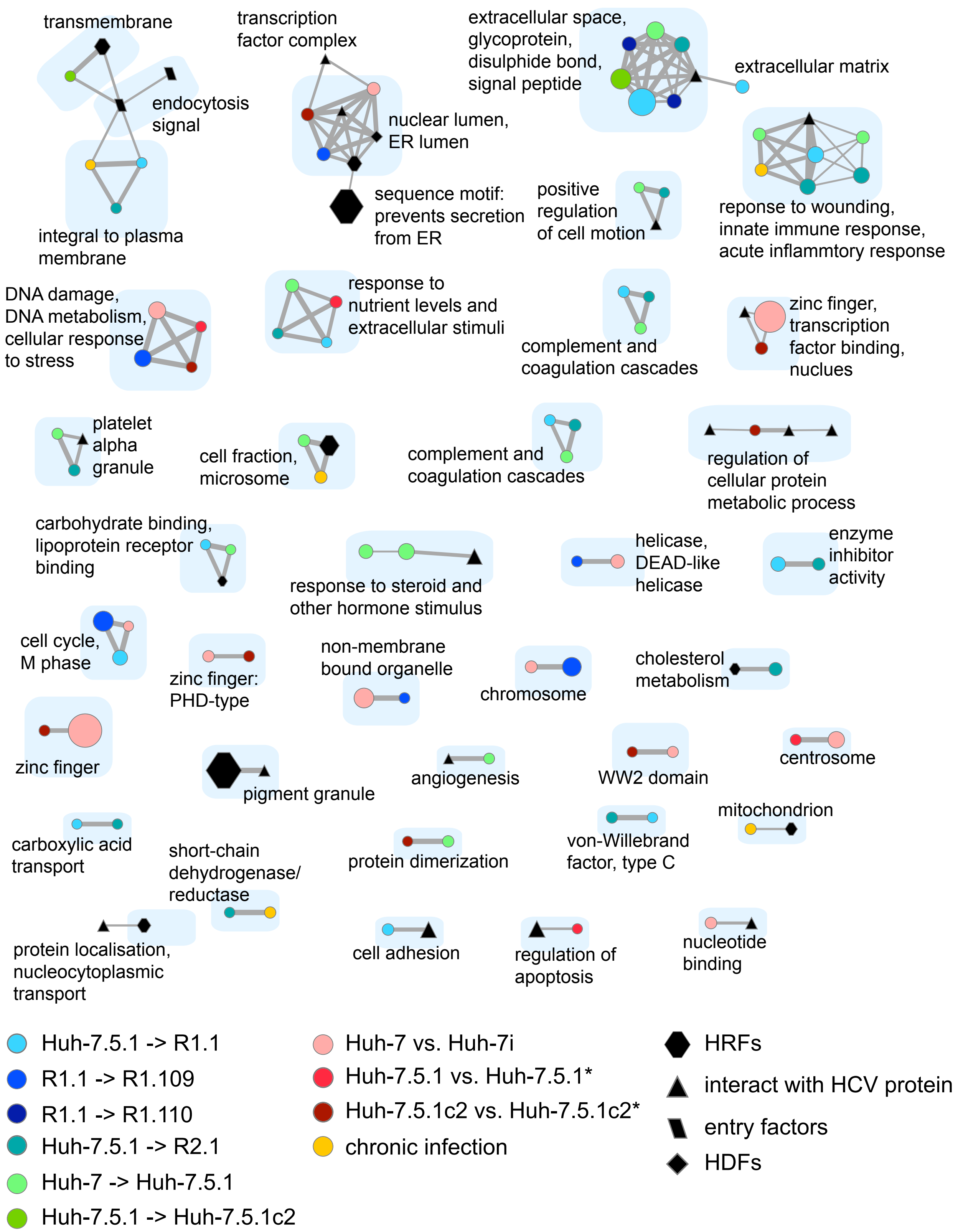
transmembrane
endocytosis signal
integral to plasma membrane
transcription factor complex
nuclear lumen, ER lumen
sequence motif: prevents secretion from ER
extracellular space, glycoprotein, disulphide bond, signal peptide
extracellular matrix
positive regulation of cell motion
reponse to wounding, innate immune response, acute inflammtory response
DNA damage, DNA metabolism, cellular response to stress
response to nutrient levels and extracellular stimuli
complement and coagulation cascades
zinc finger, transcription factor binding, nuclues
platelet alpha granule
cell fraction, microsome
complement and coagulation cascades
regulation of cellular protein metabolic process
carbohydrate binding, lipoprotein receptor binding
response to steroid and other hormone stimulus
helicase, DEAD-like helicase
enzyme inhibitor activity
cell cycle, M phase
zinc finger: PHD-type
non-membrane bound organelle
chromosome
cholesterol metabolism
zinc finger
pigment granule
angiogenesis
WW2 domain
centrosome
carboxylic acid transport
short-chain dehydrogenase/ reductase
protein dimerization
von-Willebrand factor, type C
mitochondrion
protein localisation, nucleocytoplasmic transport
cell adhesion
regulation of apoptosis
nucleotide binding
Huh-7.5.1 -> R1.1
R1.1 -> R1.109
R1.1 -> R1.110
Huh-7.5.1 -> R2.1
Huh-7 -> Huh-7.5.1
Huh-7.5.1 -> Huh-7.5.1c2
Huh-7 vs. Huh-7i
Huh-7.5.1 vs. Huh-7.5.1*
Huh-7.5.1c2 vs. Huh-7.5.1c2*
chronic infection
HRFs
interact with HCV protein
entry factors
HDFs
