# Supplementary figures and images for: An Integrated Transcriptomic and Meta-Analysis of Hepatoma Cells Reveals Factors That Influence Susceptibility to HCV Infection
Source: PLoS One. 2011 Oct 25;6(10):e25584. doi: 10.1371/journal.pone.0025584 (PMC3201949; doi:10.1371/journal.pone.0025584)

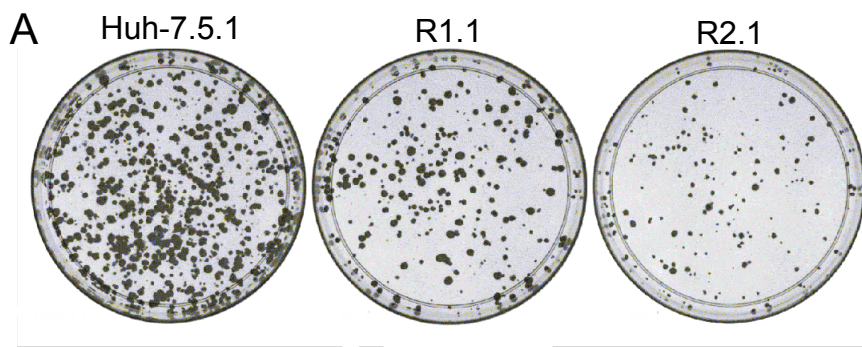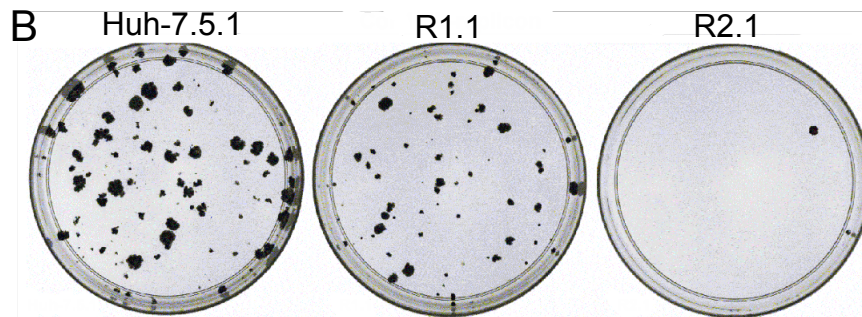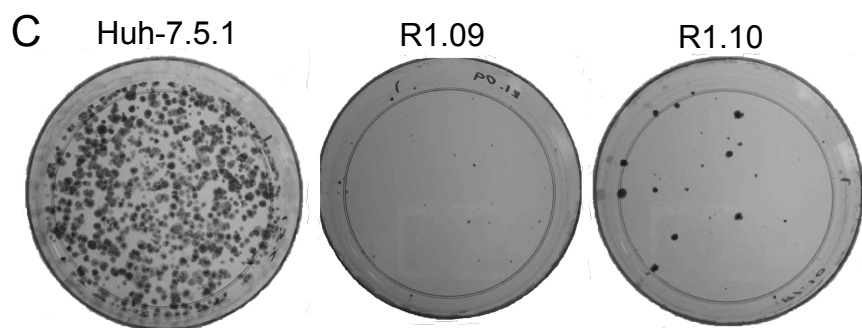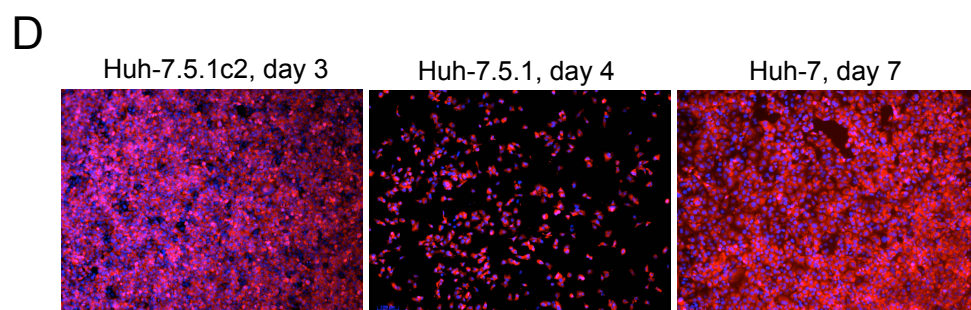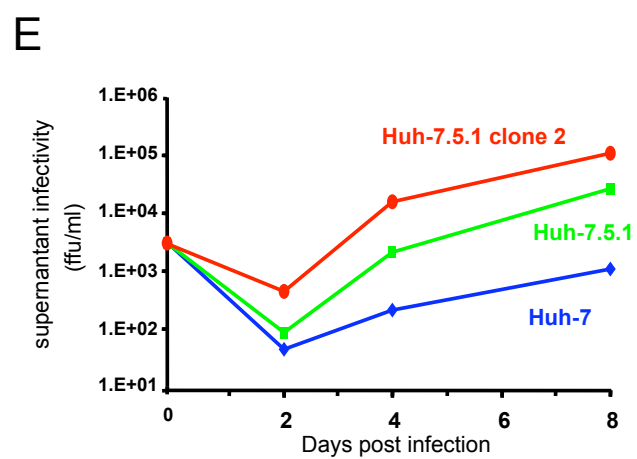

Supplement: Figure S2 — (A) Huh-7.5.1, R1.1 and R2.1 G418 resistant colonies transfected with HCV genotype 1b (Con1) subgenomic replicon encoding a neomycin resistance gene. (B) Huh-7.5.1, R1.1 and R2.1 G418 resistant colonies transfected with Con1 full-length replicon RNA encoding a neomycin resistance gene. (C) Huh-7.5.1, R1.09 and R1.10 G418 resistant colonies transfected with HCV genotype 1b (Con1) subgenomic replicon encoding a neomycin resistance gene. (D) Staining of HCV infected cells for viral E2 protein at 3 (Huh-7.5.1c2), 4 (Huh-7.5.1) and 7 (Huh-7) days post inoculation.(E) Infectivity of supernatant produced from infected cells at 2, 4 and 8 days post infection. (PDF) [file pone.0025584.s002.pdf]
