# Supplementary material for: An Integrated Transcriptomic and Meta-Analysis of Hepatoma Cells Reveals Factors That Influence Susceptibility to HCV Infection
Source: PLoS One. 2011 Oct 25;6(10):e25584. doi: 10.1371/journal.pone.0025584 (PMC3201949; doi:10.1371/journal.pone.0025584)
Supplement: Figure S3 — PCA analysis was carried out on RMA expression values of each array. Principal components 1, 2 and are plotted for each array. (PDF) [file pone.0025584.s003.pdf]

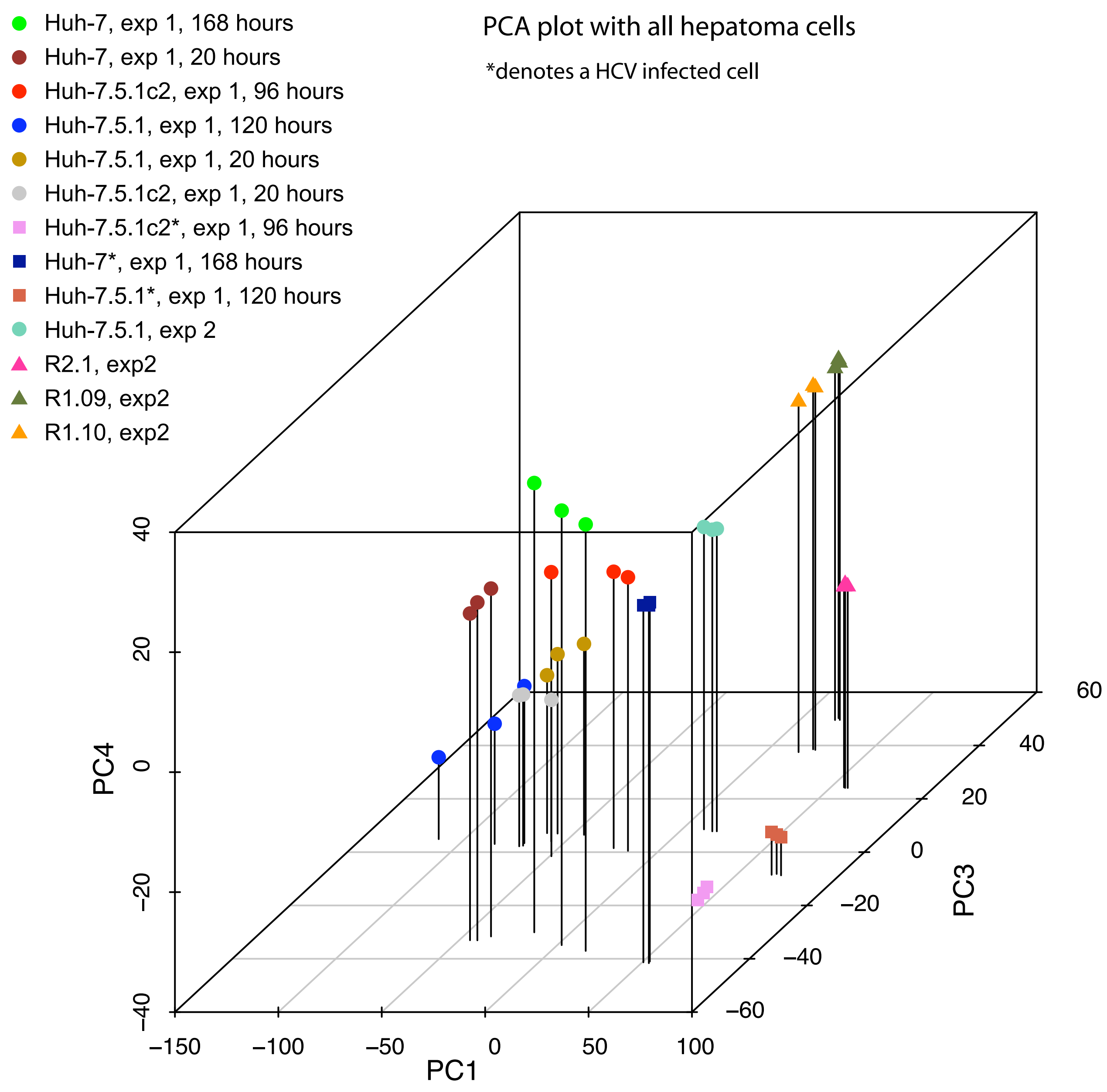

Huh-7, exp 1, 168 hours
Huh-7, exp 1, 20 hours
Huh-7.5.1c2, exp 1, 96 hours
Huh-7.5.1, exp 1, 120 hours
Huh-7.5.1, exp 1, 20 hours
Huh-7.5.1c2, exp 1, 20 hours
Huh-7.5.1c2*, exp 1, 96 hours
Huh-7*, exp 1, 168 hours
Huh-7.5.1*, exp 1, 120 hours
Huh-7.5.1, exp 2
R2.1, exp2
R1.09, exp2
R1.10, exp2
PCA plot with all hepatoma cells
*denotes a HCV infected cell
PC4
PC1
PC3
40
20
0
−20
−40
−150
−100
−50
0
50
100
60
40
20
0
−20
−40
−60
